# Musical and psychomotor interventions for cognitive, sensorimotor, and cerebral decline in patients with Mild Cognitive Impairment (COPE): a study protocol for a multicentric randomized controlled study

**DOI:** 10.1186/s12877-022-03678-0

**Published:** 2023-02-06

**Authors:** CE. James, C. Stucker, C. Junker-Tschopp, AM. Fernandes, A. Revol, ID. Mili, M. Kliegel, GB. Frisoni, A. Brioschi Guevara, D. Marie

**Affiliations:** 1 Geneva School of Health Sciences, Geneva Musical Minds Lab (GEMMI lab), University of Applied Sciences and Arts Western Switzerland HES-SO, Avenue de Champel 47, 1206 Geneva, Switzerland; 2 Faculty of Psychology and Educational Sciences, University of Geneva, Boulevard Carl-Vogt 101, 1205 Geneva, Switzerland; 3 Geneva School of Social Work, Department of Psychomotricity, University of Applied Sciences and Arts Western Switzerland HES-SO, Rue Prévost-Martin 28, 1205 Geneva, Switzerland; 4 Faculty of Psychology and Educational Sciences, Didactics of Arts and Movement Laboratory, University of Geneva, Switzerland. Boulevard Carl-Vogt 101, 1205 Geneva, Switzerland; 5 Faculty of Psychology and Educational Sciences, Center for the Interdisciplinary Study of Gerontology and Vulnerability, University of Geneva, Switzerland, Boulevard du Pont d’Arve 28, 1205 Geneva, Switzerland; 6 University Hospitals and University of Geneva, Memory Center, Rue Gabrielle-Perret-Gentil 6, 1205 Geneva, Switzerland; 7 Leenaards Memory Center, Lausanne University Hospital, Chemin de Mont-Paisible 16, 1011 Lausanne, Switzerland; 8 CIBM Center for Biomedical Imaging, MRI HUG-UNIGE, University of Geneva, Geneva, Switzerland

**Keywords:** Mild cognitive impairment, Randomized controlled trial, Non-medical interventions, Cognitive performance, Sensorimotor performance, Experience induced functional and structural brain plasticity, Music instrumental practice, Psychomotor therapy, Passive control group, Multivariate data-driven analyses

## Abstract

**Background:**

Regular cognitive training can boost or maintain cognitive and brain functions known to decline with age. Most studies administered such cognitive training on a computer and in a lab setting. However, everyday life activities, like musical practice or physical exercise that are complex and variable, might be more successful at inducing transfer effects to different cognitive domains and maintaining motivation. "Body-mind exercises", like Tai Chi or psychomotor exercise, may also positively affect cognitive functioning in the elderly. We will compare the influence of active music practice and psychomotor training over 6 months in Mild Cognitive Impairment patients from university hospital memory clinics on cognitive and sensorimotor performance and brain plasticity. The acronym of the study is COPE (Countervail cOgnitive imPairmEnt), illustrating the aim of the study: learning to better "cope" with cognitive decline.

**Methods:**

We aim to conduct a randomized controlled multicenter intervention study on 32 Mild Cognitive Impairment (MCI) patients (60–80 years), divided over 2 experimental groups: 1) Music practice; 2) Psychomotor treatment. Controls will consist of a passive test–retest group of 16 age, gender and education level matched healthy volunteers.

The training regimens take place twice a week for 45 min over 6 months in small groups, provided by professionals, and patients should exercise daily at home. Data collection takes place at baseline (before the interventions), 3, and 6 months after training onset, on cognitive and sensorimotor capacities, subjective well-being, daily living activities, and via functional and structural neuroimaging. Considering the current constraints of the COVID-19 pandemic, recruitment and data collection takes place in 3 waves.

**Discussion:**

We will investigate whether musical practice contrasted to psychomotor exercise in small groups can improve cognitive, sensorimotor and brain functioning in MCI patients, and therefore provoke specific benefits for their daily life functioning and well-being.

**Trial registration:**

The full protocol was approved by the Commission cantonale d’éthique de la recherche sur l'être humain de Genève (CCER, no. 2020–00510) on 04.05.2020, and an amendment by the CCER and the Commission cantonale d'éthique de la recherche sur l'être humain de Vaud (CER-VD) on 03.08.2021. The protocol was registered at clinicaltrials.gov (20.09.2020, no. NCT04546451).

**Supplementary Information:**

The online version contains supplementary material available at 10.1186/s12877-022-03678-0.

## Background

### Normal aging and cognitive decline

In the context of a rapidly growing elderly population, interventions that may slow down cognitive decline and support the maintenance of independence in the elderly are crucial for senior care and determining appropriate social, economic, and public health management. Age-related cognitive decline impacts particularly processing speed, episodic and working memory, visuospatial skills, executive functions, and fine and gross motor skills [1, 2] and is associated with global cerebral atrophy (brain degeneration). However, some regions, such as the prefrontal cortex and the hippocampus, are more affected than others [3, 4].

Executive functions are one of the cognitive domains most affected by aging [5, 6], and especially so in MCI patients [7–12].

### Training regimens to countervail age-related cognitive decline

Evidence suggests that regular cognitive training [13–15] can boost or maintain cognitive and brain functions known to decline with age. Such cognitive training is most often administered on a computer and in a lab setting and far transfer effects to non-trained domains are scarce [16–19]. Yet, training regimens that can be integrated into activities of daily living, such as musical practice [20, 21], dancing [22], playing chess [23] or physical exercise [24–27], might be more effective. Their complex and variable nature may induce transfer effects to different cognitive domains more efficiently [28, 29], and moreover, increase motivation [4, 25, 30], allowing maintaining these activities in the long term (over several years). However, only a direct comparison of regimens that can be integrated into daily activities versus training in a laboratory setting (computerized or not), of comparable duration and intensity, with a number of patients providing sufficient statistical power, could yield a well-founded answer to this presumption. This should be investigated in future research.

Instrumental musical training seems to confer certain advantages over other types of exercise [21, 31–33]. Playing an instrument directly involves both core cognitive components, such as working memory, attention, processing speed, and executive functions, and also abstract reasoning [34]. The multimodal nature of musical performance may represent one of the explanations of its efficacy, as auditory, sensorimotor, proprioceptive, and visual feedback are put into relationship with the expected acoustic musical end product and also with the music score to correct and refine the motor performance [35], interconnected by memory functions, executive functions and abstract reasoning [36].

Moreover, the auditive aspect is unique to music-making and processing. Among all sensory systems, the auditive one is the fastest [37]. This ultrafast processing in a dynamic setting may thus drive the subcomponents of WM updating, i.e. temporal sequencing and monitoring [38].

Then, in contrast to gross motor functions implicated in psychomotor therapy, fine sensorimotor skills play a crucial role in playing a musical instrument, including bimanual coordination.

Finally, music-making's emotional and rewarding aspects represent an intrinsic motivator underpinned by neurochemistry (e.g., dopamine release) [39–41]. Musical activities also impact other physiological processes, inciting potential positive effects on the cardiovascular system (cortisol decrease) and increasing stamina (dopamine increase), via neurochemistry [39, 41, 42].

Musical training performed during youth or midlife positively influences cognitive reserve (resilience to neuro-pathological damage in older adults) [43–45], and may delay the onset of dementia [46, 47]. Improved executive function, well-being, visual memory, and hearing in noise manifested in healthy older adults after musical training starting at an advanced age [20, 21, 33, 48]. Several recent studies [49–52] could show advantages following musical training after retirement in healthy older adults, associated with brain changes, for certain capacities implied in activities of daily living (speech in noise perception, verbal memory, and manual dexterity). Musical training thus seems a promising approach.

Physical training also represents a favorable approach in the fight against pathological decline, defined as a loss of cognitive capacities beyond normal aging [53]. Aerobic training positively influences cognitive function in the elderly [25–27, 54]. It may even retard dementias like Alzheimer's disease [55]. Although less acknowledged, cognitive and sensorimotor benefits also showed following non-aerobic exercises, like Baduanjin, Tai Chi, or psychomotor training [56–58], that instead involve slow non-aerobic movement. Yet this type of physical intervention seems better suited for frail older adults than aerobic exercise.

Recent evidence shows that psychomotor treatment, pertaining to the so-called body-mind approaches, may also positively influence physical, cognitive, and emotional functioning in healthy elderly [59]. A 6-months Randomized Controlled Trial (RCT) using Norwegian physical psychomotor therapy conducted in 105 adults (18–70 years old) [60] showed considerable improvement of health-related quality of life evaluated by questionnaires (physical functioning, general and mental health, emotional issues, social functioning, self-esteem, etc.). Notably, in healthy elderly, psychomotor interventions induced cognitive (processing speed, executive functioning, and attention) and physical benefits for gross motor control (improvements in balance, gait, and mobility), after only 10–12 weeks of training [58]. The latter is vital as fall risk increases in MCI patients [61]. Like musical practice, psychomotor practice thus represents a good candidate for non-medical interventions aiming to countervail normal and pathological mental decline in the elderly.

Two RCTs applying musical interventions on MCI patients [62, 63], showed positive effects on verbal fluency, attention, executive functions and praxis (Clock Drawing test), on a general cognitive test (MMSE; Mini-Mental State Examination) [64] and mood. The musical training involved vocal and instrumental improvisation and was compared to soft gymnastics training (active control condition); both lasted 3 months. The control group, however, received much shorter training sessions. Two recent systematic reviews [65, 66] showed a significant positive effect of active music-making and other music-based interventions on cognitive functioning in patients with MCI and dementia.

A recent review by Cespon et al. [67] postulates that MCI patients profit most from multimodal programs, combining cognitive and physical stimulation. Cespon et al. divide non-invasive interventional approaches into cognitive interventions and physical exercise. The 2 non-aerobic interventions proposed here comprise elements of both and thus constitute such multimodal activities. Psychomotor exercise involves gross motor control (gait, balance, mobility) and also stimulates cognitive functions. Music instrumental practice taps directly into cognitive functions, particularly executive functions, but also drives fine motor control (manual dexterity, bimanual coordination, upper limb resistance), and neurochemistry.

Concerning brain plasticity, 2 recent RCT trials used structural and functional Magnetic Resonance Imaging (MRI) to assess the efficacy of treatments on MCI patients [68, 69]. Simon and colleagues [68] compared mnemonic strategy training over 2 weeks to an active control group of MCI patients who followed an educational program. Neuropsychological tests and functional MRI (fMRI), the latter using a Face-Name Recognition Task, were measured before and after training. MCI patients in the training group showed improved face-name memory and higher fMRI activation in the left anterior temporal lobe. No indication of far transfer occurred. The experimental group's face-name memory task demonstrated preserved improvement after one and three months. The "Train the Brain" consortium [69] compared the effects of combined 7-month physical-cognitive training to a passive control group on cognitive decline, gray matter volume, cerebral blood flow (CBF) in hippocampal areas, and fMRI activity elicited by a cognitive task. Cognitive status significantly improved in the training group whereas it decreased in the control group. In the training group, parahippocampal CBF increased. Both studies [68, 69] show that non-pharmacological interventions may improve cognitive functioning and brain health in MCI patients.

Mild Cognitive Impairment (MCI) represents a cognitive state between normal aging and dementia, with a minor age-related loss of cognitive ability that does not importantly impact daily life functioning. Symptoms most frequently involve impairments in memory, attention, spatial orientation, executive functions [70–72]. Increased fall risk is an associated condition [61].

Although some MCI patients may stay stable or even recover to some extent [73], some of them are on a downward slope to overt dementia, i.e., a pathway of no return [73, 74].

### Main hypothesis

We anticipate that, compared to changes in the healthy control group, 6 months of intensive musical practice or psychomotor training will distinctively reinforce general cognitive and physical functioning of MCI patients. Overall, we anticipate that musical instrument practice will have a significantly more profound impact.

### Theoretical framework

Our approach is based on an emerging field of evolutionary medicine investigating the effects of lifestyle on health and well-being [75]. Apparently, moderate intensities of physical exercise with a simultaneous cognitive load seem associated with human evolutionary history [76]. The adaptive capacity model (ACM) [76], presumes that lifestyle changes, which combine the benefits of moderate physical exercise and novel cognitive challenges may provoke neuroplasticity most strongly and therefore provide neuroprotection against aging. Musical practice and psychomotor training both comprise such a combination of moderate-intensity physical with cognitive training. Moderate-intensity physical exercise seems to provide the strongest cognitive benefits in humans [77]. This neuroprotection, potentially resulting in an increase of cognitive reserve or resilience, may then contribute to psychological well-being (mental health) [78]. More research is needed to confirm this postulate [76].

### Goals

With this project, we hope to generate new scientific knowledge on the effectiveness of non-medical therapies that involve moderate physical exercise and cognitive stimulation, to improve the mental, physical and cerebral health of MCI patients.

If the results turn out positive, the autonomy, well-being, and quality of life of these patients and their caregivers might be enhanced, and the risk of developing Alzheimer's disease or another type of dementia delayed or reduced.

If so, the study could serve as a springboard for developing and implementing innovative, targeted musical and psychomotor or other non-medical interventions for different types of pathological populations with cognitive impairment in the context of population aging. In addition, these interventions will be less costly than more traditional treatments.

### Rationale

Cognitive decline represents a major threat among the deleterious effects of population aging. We propose to conduct an RCT on MCI patients and investigate whether intensive musical or psychomotor group interventions can improve their cognitive and sensorimotor functioning and induce brain plasticity compared to a passive control group of healthy matched individuals.

Longitudinal applied neuroscience studies on non-medical interventions are still rare, although particularly interesting in the early stages of pathological decline, when a reverse tendency may still be provoked. Moreover, by incorporating brain data, we can gain a more comprehensive understanding of those early-stage mental disorders and their potential development.

To optimize the learning process, we offer intensive training regimens: music practice and psychomotor treatment interventions take place twice a week over 6 months, provided by professionals in each field (holding a Master degree), completed by daily homework.

To accurately monitor development, assessments take place before (baseline), at mid-term and immediately after the interventions. Cognitive and sensorimotor development is assessed through a battery of psychometric tests, including an evaluation of well-being and autonomy in daily life. To evaluate concurrent brain plasticity, functional and structural Magnetic Resonance Imaging (MRI) measurements are performed at the same time points.

### Aims

This RCT aims to determine if 2 specifically developed innovative behavioral non-medical experimental interventions over 6 months, i.e. intensive musical instrumental practice and psychomotor interventions in small groups may have an important societal impact, via the reduction or stabilization of cognitive, sensorimotor and cerebral decline, in MCI patients compared to a passive control group, composed of healthy matched controls (from now on: passive control group).

### Hypotheses and outcomes

### Main hypothesis

We hypothesize that 6 months of intensive musical practice or psychomotor training will improve or stabilize -each in their own way- general cognitive and physical functioning of MCI patients, compared to the passive control group. Altogether we expect a far stronger effect of musical instrumental practice.

### Comparisons

To evaluate our outcomes, we will compare the development in each experimental group of MCI patients over time, to the development in the passive control group over time. We will also compare the development over time between the 2 experimental groups (see the section Specific outcomes according to each intervention). As we are interested in development, we will incorporate the baseline scores in our statistical models, to correct for differences at baseline. All groups, the passive control group included, may show test–retest effects over time.

### Primary Outcome

The primary outcome is increase of the total weighted score at the COGTEL test^1^ [79, 80] at mid-term, after 3 months (t1) and after 6 months (t2) of interventions in the musical practice group and/or the psychomotor practice group of MCI patients compared to baseline and to the passive control group.

The COGTEL served as primary outcome in several recent clinical and epidemiological studies [81–85].

### Choice of the COGTEL

Although the COGTEL weighted score and the Mini-Mental State Examination (MMSE) [64] correlate at 0.93 [80], the COGTEL covers more cognitive domains (n = 6) than traditional screening tools like the MMSE, which is rather a dementia screening. It comprises 6 subtests covering prospective memory, short- and long-term verbal memory, working memory (digit span), verbal fluency and inductive reasoning.

The COGTEL thus allows a more fine-tuned analysis of cognitive abilities [86]. In addition, test–retest reliability is good (r = 0.85) [80].

### Secondary hypotheses

1. We hypothesize that the 2 experimental interventions over 6 months in MCI patients, will improve basic cognitive and executive functioning, compared to the passive control group that will stay stable or regress.
2. We hypothesize that the 2 experimental interventions over 6 months in MCI patients, will improve sensorimotor functioning, compared to the passive control group that will stay stable or regress.
3. We hypothesize that functional and structural brain plasticity associated or not with cognitive and sensorimotor benefits will occur over 6 months in the 2 experimental groups of MCI patients, compared to the passive control group that will stay stable or regress.
4. We hypothesize that daily living activities will improve over 6 months in the 2 intervention groups of MCI patients, compared to the passive control group that will stay stable or regress.
5. We hypothesize that subjective well-being will improve in the 2 intervention groups of MCI patients over 6 months, following the regular social interactions and structured activities during the interventions and at home [87], compared to the passive control group that will stay stable or regress.

### Secondary Outcomes

1. Improve basic cognition and executive function functioning in the 2 experimental intervention groups of MCI patients over 6 months, compared to the passive control group, measured by the scores of the following cognitive tests: individual subtests of the COGTEL [79, 80], d2-R test [88, 89], Trail Making tests [90], Go/No-Go [91], visual working memory (fMRI test) [92] and Clock Drawing test^2^ [93].
2. Improve sensorimotor functioning in the 2 experimental intervention groups of MCI patients over 6 months, compared to the passive control group, measured by the individual and total scores of the sensorimotor tests: Clock Drawing test [93], Purdue Pegboard [94], Unipedal balance test [95] and Laterality recognition test [96].
3. Induce functional and structural brain plasticity and connectivity associated or not with cognitive and sensorimotor benefits in the 2 experimental intervention groups of MCI patients over 6 months, compared to the passive control group, measured by structural, resting-state functional, functional and DTI (Diffusion Tensor Imaging) MRI.
4. Improve daily living activities as measured by the Amsterdam Instrumental Activity of the Daily Living Questionnaire [97] in the experimental groups of MCI patients over 6 months, compared to the control group
5. Improved subjective well-being in the 2 experimental groups of MCI patients after 6 months, compared to the control group, measured by the World Health Organization Quality of Life-BREF questionnaire (BREF for abbreviated) (WHOQOL-BREF) [98].

### Specific outcomes according to each intervention 

#### Music practice intervention

Instrumental musical training seems to confer advantages over other types of training in children [32, 99] and elderly [21, 33] and specifically so in MCI patients [62, 65]. Playing an instrument requires both core cognitive components, such as working memory, attention, or processing speed [20, 99, 100], and complex components such as executive functions and even abstract reasoning [20, 21, 34, 101]. Therefore we expect greater advantages in the music group compared to the psychomotor group for the weighted COGTEL score, individual subtests of the COGTEL [79, 80], d2-R test [88, 89], Trail Making tests [90], Go/No-Go [91], visual working memory (fMRI test) [92] and Clock Drawing test [93] (corresponding to Secondary Outcomes no. 1). In addition, sensorimotor function and coordination of the upper body and the hands play a major role and may increase in this group as compared to the psychomotor and control group (Clock Drawing test), Purdue Pegboard test [101, 102]. Emotion regulation may also be enhanced [103], and indirectly impact executive functions.

Functional and structural brain plasticity associated with cognitive and sensorimotor benefits may show in the prefrontal cortex [104–106], the hippocampus [4, 34, 107–109] and sensorimotor areas [110–114], including the auditory cortices [104, 115–117] and also by enhanced functional and structural connectivity [50, 51, 118, 119].

#### Psychomotor intervention

Cognitive benefits such as reinforced processing speed, attention, and executive functions [58, 59, 120] but also emotion regulation [121] and visuospatial memory [120] may occur in this group, but we expect less strong benefits than in the music group for the weighted COGTEL score, individual subtests of the COGTEL [79, 80], d2-R test [88, 89], Trail Making tests [90], Go/No-Go [91], visual working memory (fMRI test) [92] and Clock Drawing test [93] (Secondary Outcomes no. 1). However, whole body sensorimotor function, balance, and body schema representation may improve more strongly following psychomotor interventions, compared to the music practice and the control group, as was shown for coordination training, Pilates and other body-mind approaches [120, 122, 123], and specifically for psychomotor training [58, 124]. Therefore, scores on the Unipedal balance test, and the Laterality recognition test may be enhanced compared to the music group. As no studies exist measuring brain plasticity following psychomotor training, we can not specify brain plasticity effects citing the literature. Still, they may overlap to some extent with those observed for music practice, concerning the prefrontal cortex and sensorimotor areas. Sensorimotor activity will more likely implicate areas that involve the whole body, and act on associated functional and structural connectivity [125].

## Methods & design

Our study consists of an extension of the study by Biasutti and Mangiacotti, 2018 [62] on music practice in MCI patients as compared to soft gymnastics. However, we will opt for a more comprehensive approach changing the following elements with respect to the experimental design of Biasutti and Mangiacotti, 2018: 1) our psychometric measures will not only involve cognitive tasks, but also sensorimotor performance, activities of daily living and well-being, 2) importantly, in addition, we will collect different functional and structural brain imaging data, 3) instead of soft gymnastics as a control group, we added psychomotor practice, a body-mind approach, as a second experimental intervention, 4) our study will be more extended over time (6 months against 3 months for [62]), and 5) both experimental interventions strongly involve sensorimotricity, often weakened in this population [61, 72], 6) in the current study, we will essentially train the music groups to learn and produce existing musical excerpts together, thus enhancing memory functions, although some improvisation will be included in the interventions, 7) our intervention groups will be small (n = 3–4, against n ~ 20 in [62]), permitting sufficient personal attention from the teachers for each participant, and meanwhile allowing group dynamics in order to strongly implicate executive functions (inhibition, working memory, planning, etc.) and attention, that are key targets for stimulating cognitive reserve, 8) comparing psychomotor to music practice interventions will allow to disentangle whole body sensorimotor from hand and arm sensorimotor aspects, and to detect domain specific cognitive and emotion related effects of each regimen, 9) a passive healthy control group will control for age-related natural brain atrophy and test–retest effects of the psychometrics and fMRI tasks.

Thus, the current study introduces numerous novel features. Please see the section "Innovative Aspects” in the Discussion Section for a comprehensive overview.

### Design, interventions and setting

### Study design

The proposed study is a 1) block-randomized, 2) 3-arm, 3) multicentric (HUG^3^, Genève; CHUV^4^, Lausanne), 4) national (Switzerland), 5) intervention study.

Blocked randomization is applied to the experimental groups only, blocking for the factors 1) age, 2) gender, 3) COGTEL weighted score [80] and 4) MCI diagnosis (1. amnestic; 2. non-amnestic) to balance both intervention groups for those factors. Experts at the memory clinics will select MCI diagnosed patients for participation.

We use a three-arm design: 2 experimental groups of MCI patients, and one matched passive healthy control group. Except at the level of statistical analyses, no blinding is applied.

The blocked (or stratified) randomization procedure is performed at each site separately, aiming to achieve an equal number of music and psychomotor groups at each site, using Ward’s Hierarchical Agglomerative Clustering Method [126]. We will use this method to establish duplets that are maximally similar with respect to the 4 blocking factors, based on minimum Euclidean distance. Subsequently, an algorithm will attribute randomly one participant of each duplet to each experimental group. So, half of the participants will end up in the music practice intervention (n ~ 16), and the other half in the psychomotor intervention (n ~ 16). A control group of matched (age, gender, education level) healthy controls (n ~ 16) that are not part of the randomization process, will be recruited in Geneva, and will receive financial compensation (gift vouchers).

In the documentation that we will provide to the patients we mention that there are 2 different intervention groups that we want to compare for their effectiveness to improve cognitive and motor function, and that for scientific reasons the repartition to the groups will be determined by chance. We will also mention that if patients refuse to accept their group allocation, they cannot participate in the study.

The psychometric battery consists exclusively of validated tests. Cognitive screening at the HUG and the CHUV is similar. The HUG uses the MMSE score [64] and the Hospital Anxiety and Depression Scale (HADS) [127]. At the CHUV the test used for general cognition is the Montreal Cognitive Assessment (MoCA) [128, 129]; they also use the HADS.

We will cope with test–retest effects by 1) using different versions, or different items, or different order of items of the tests at t0 (baseline, before the interventions), at t1 (after 3 months of interventions) and at t2 (after 6 months of interventions), whenever possible and 2) taking into account the test–retest effects in the healthy passive control group between timepoints.

### Interventions

Both experimental conditions consist in 2*45 min of group interventions per week over 6 months (~ 40 sessions in total) in small groups (n ~ 3–4) plus short daily exercises to perform at home (~ 30 min/day, 4* per week). The passive control group will not receive any intervention and only take part in the measurements.

### Experimental condition / Intervention 1: musical practice group intervention

The musical practice group will receive interventions with steel "tongue drums", provided by a therapist (a professional musician at MSc level) who has experience working with elderly and patients in groups. Different styles of music are played ("from Bach to the Beatles"). Steel tongue drums are modern percussion instruments of the idiophone family, relatively small (the ones we use are 12 inches / 30.5 cm) and thus easily manageable. They are delivered with a small bag for transportation, and each participant will receive an instrument to take home for the duration of the intervention. The instruments we chose dispose of 13 half-tones or minor seconds, thus composing together a full chromatic octave plus one note (an octave above the lowest note). These instruments can be played with the hands and fingers or with drumsticks. Their advantages are that they provide strong sensorimotor feedback, and that their tonal range allows producing melodies and harmonies together. Then, they generate a pleasant mellow sound. They are low cost and very suitable for group play. During the interventions, individual and group instruction, also with accompaniment by the teacher, are alternated. The therapist assigns homework encompassing musical exercises and rehearsal of musical pieces learned during the lessons to be performed daily (~ 30 min/day). A simple form of music notation using numbers and recordings will assist the patients during homework. Classical music notation may be used after t1, according to group level.

### Experimental condition / Intervention 2: psychomotor treatment group intervention

Based on a holistic vision of the human being, the psychomotor approach targets the interdependence between sensorimotor, cognitive, and emotional development. This body-mind training focuses on movement, particularly on developing the body schema. It proposes activities like Tai Chi, involving slow controlled movements, but is more inward-oriented. Psychomotor treatment involves interactions between physical, emotional and cognitive processes, aiming to enhance body-awareness.

Body exploration through conscious auto massage, tonic regulation exercises, balance, posture, and movement exercises are performed either with open or closed eyes. Emotion mime and recognition are part of the interventions. The incorporation of the entire body in game or drama settings, as well as postural imitation exercises, round out this training regimen. The sensory and emotional sensations following the exercises are discussed with the patients to enhance cognitive representation. This approach is used in various pathological contexts. A certified psychometrician, experienced in working with cognitively impaired elderly, will provide the interventions. Workouts performed during the interventions and simple exercises explained during the training should be performed at home daily (~ 30 min/day).

### Passive control condition

In this longitudinal study, the healthy participant group serves as a control for age-related natural brain atrophy and test–retest effects at the psychometric battery and fMRI tasks.

There is an additional reason for adopting a healthy control group: one of the university hospital memory clinics where we recruit our patients does not accept active or passive control groups of patients in non-drug intervention studies, because they provide no benefit to the patients. The healthy passive control group participants must adhere to the same inclusion and exclusion criteria as the experimental groups, except for an MCI diagnosis. They will be assessed in the same way as the experimental groups (battery and MRI measurements at t0, t1 and t2). Control participants will be matched to the experimental groups for age, gender and education level.

### Setting

For Geneva participants, music interventions and passage of the behavioral test battery take place at the Geneva School of Health Sciences (Haute Ecole de santé de Genève (HEdS-GE) of the University of Applied Sciences and Arts Western Switzerland (HES-SO)). The psychomotor training takes place at the Department of Psychomotricity (Haute école de travail social de Genève, HETS-GE) of the University of Applied Sciences and Arts Western Switzerland HES-SO, Geneva School of Social work. Brain imaging (MRI) is performed at the BBL, the Brain and Behaviour Laboratory at the University Medical Center of the Geneva University.

For Lausanne participants, all interventions and passage of the behavioral test battery take place at the Leenaards Memory Center of the CHUV, and brain imaging (MRI) will be performed at the Laboratoire de recherche en neuroimagerie (LREN; Laboratory for Research in Neuroimaging) in the same building, also at the CHUV.

### Study population

#### Inclusion and exclusion criteria

### Inclusion criteria

1. MCI diagnosis by experts at the memory clinics; 2. MMSE score (Mini-Mental State Examination) [64] ≥ 23 or MoCA ≥ 17 [130, 131]; 3. Hospital Anxiety and Depression Scale (HADS) [127] < 14 (< 7/21 for anxiety and < 7/21 for depression); 4. Age between 60 and 80 years; 5. Right-handedness [132]; 6. Fluent in French; 7. Able to give informed consent as documented by signature.

Only right-handed participants will be included, because a minority of left-handed individuals show a different hemispheric organization, e.g. for language and music functions [133–135]. Left-handed participants could induce variability in the data and diminish experimental effects. Right-handed persons represent more than 90% of the population.

### Exclusion criteria

1. Serious motor deficits; 2. Impaired/not-corrected hearing 3. Serious physical and mental comorbidities; 4. Participation in physical or cognitive training over the last 12 months; 5. Maximum 5 years of official music education over the lifespan outside the school curriculum or during the last 3 years; 6. Intensive physical activity over the last 12 months (sports or body-mind exercises); 7. Left-handed or ambidextrous; 8. MRI incompatibility (claustrophobia, cardiac stimulator, implants…).

### Recruitment, prescreening and informed consent procedures (experimental groups)

The recruitment takes place at the Memory Center of the HUG (MC-HUG) in Geneva, and at the Leenaards Memory Center of the CHUV (LMC-CHUV) in Lausanne. Diagnosis of MCI is performed by specialized neurologists of the MC-HUG and the LMC-CHUV. At each site, a collaborator will call the patients who correspond to the profile and ask if they would like to engage in a 6-month intervention that includes either musical or physical training twice a week, as well as brain and behavior measures that will be repeated 3 times. The patients who manifest an interest in participating in the study will receive a letter to confirm the information exchange with the MC-HUG or the LMC-CHUV. They will be contacted by phone several days later by the clinical psychologist of the research team to perform a more thorough check concerning the inclusion/exclusion criteria and to provide more extensive information on the interventions and the randomization procedure.

If the patient seems compatible with the criteria and interested in participating, the declaration of informed consent, a summary information sheet and a series of questionnaires (demographic, cognitive reserve index, daily living activities, musical activities, physical activities & stressful life events; for details and references, see the section Measurements and Procedures and Supplementary Table 1) will be sent to their home address to fill in alone or with a caregiver. After reading the information sheet, before filling in any questionnaire, the patient/participant should sign the declaration of informed consent. During the first laboratory visit, the clinical psychologist of the research team will first countersign the declaration of informed consent and then check and finish completing the questionnaires, together with the patient, at the HEdS-GE or LMC-CHUV (see Supplementary Table 1**)**. By doing so, the psychologist verifies in detail whether all inclusion and exclusion criteria are met.

During the first lab visit (see Supplementary Table 1, Prescreening step 3), each potential participating patient will be asked to bring their signed informed consent that will be countersigned in his/her presence by the clinical psychologist. The participating patient will then receive more precise oral and written information on the study procedure. The documentation is adapted to the patient population to facilitate understanding. The information will cover the prescreening (inclusion/exclusion criteria) and its rationale, the randomization into 2 intervention groups, the pre (t0), intermediate (t1) and post-intervention (t2) behavioral testing (psychometric battery) and MRI recordings, and their potential inconveniences (fatigue, noise, etc.). If the patient still agrees to participate, and all information is clear, and if they meet all the inclusion/exclusion criteria, the informed consent can be considered validated. If not so, the informed consent form and all information on the patient will be destroyed.

A copy of the signed informed consent will be given to the participating patients.

### Consent (all groups, control group included)

Consent to voluntarily participate in the study implies: the right to withdraw at any time, consent to the use of the collected data for scientific and educational purposes (provided that the data remain anonymous) and finally, to inform the participants directly or their treating physician, in case of unexpected potential detection of abnormalities.

### Documents and information provided to the participants

In chronological order the patients/participants will receive the declaration of informed consent, a summary information sheet, extensive oral information during the first lab visit, a trust agreement^5^ to sign, a MRI safety questionnaire to be filled in before each MRI measurement, a homework questionnaire (weekly report on estimated time spent on homework), and an authorization for use of image (on a voluntary basis), as we will in principle film the interventions once per month in order to perform qualitative analyses on the learning process.

### Compensation for the MCI patients (experimental groups)

The compensation for the MCI patients consists in 6 months of free interventions, and reimbursement of transportation costs with the Geneva or Lausanne public transport system (insofar as possible).

### Recruitment, practical details

The recruitment should have started for the first wave of patients in May 2020. Finally, the COVID-19 epidemic caused a 4–5 months delay in the original schedule, and we started recruitment in September 2020 lasting till November 2020.

Mainly because of the anxiogenic and restrictive context of COVID-19, but also for personal reasons (too much time involved, etc.), most patients that seemed to fulfil the criteria initially contacted by the MC-HUG finally chose not to participate. In the end, only 14 patients that met all criteria consented to participate, and of those 4 more interrupted their participation, 2 who went into early dementia, one quit for health reasons and a last individual for personal reasons. Ten patients followed the interventions and passed the measurements, which is not enough for valid analyses (see the section Statistical power of the study).

Therefore, we recruited a second wave of patients at the LMC-CHUV, participating in the study from November 2021 to July 2022 (prescreening, interventions, and all measurements), and a third wave participating between July 2022 and April 2023. We intend to recruit enough patients to bring the total to 16 for both experimental groups. Baseline measures (t0) for the second wave started in December 2021, and for the third wave from August 2022, after the inclusion process (pre-screening and first visit).

### Recruitment and procedure for the passive control group

Healthy passive controls will be recruited in the Geneva area among community-dwelling elderly from February 2022, through advertisements in local (elderly) journals, elderly centers (flyers, posters), and on the internet (i.a. www.planetesante.ch), once the recruitment of the experimental groups is over, to allow matching with the experimental groups.

Participants will be informed about the study's aims and the role of a control group and must, in principle, consent to pass all 3 measurement points (t0, t1, t2). Except for the MCI diagnosis, the same inclusion/exclusion criteria apply to this group as to the experimental groups. They will receive financial compensation by means of gift vouchers. We will first recruit a larger pool than necessary, to allow matching to the patient group.

### Duration of the study for each patient

For the experimental groups, the pre-screening period and baseline (t0) testing will take place within approximately 6 weeks before the onset of the interventions. Both intervention groups will receive ~ 40 interventions of 45 min, 2 per week, divided over 6 months. Mid-intervention testing will take place approximately after 3 months of intervention and the post-intervention testing within a maximum of 4 weeks after the last intervention.

The total duration for an average patient to participate in the study therefore takes approximately 9 months, within a period stretching from November 2020 till July 2021 for the first wave of patients and from November 2021 till July 2022 for the second wave. For the third wave the period will extend from July 2022 to April 2023.

For the control group prescreening and baseline measurements will start in March–April 2022, the t1 measurements will take place in June-July, and the t2 measurements in September–October 2022.

### Potential biases concerning patient recruitment and attrition

Potential biases could reside in the long-lasting absence of patients during the interventions due to illness, vacation, or family reasons. We will cope with attrition as follows. 1) Patients will sign a trust agreement aiming not no miss more than 10% of the interventions and not to engage in any supplementary activities similar to those proposed in this study. This trust assignment is relevant only if patients continue to participate in the interventions; it is null and void if the patient withdraws from the research. 2) The team will temporarily reorganize intervention groups within the same experimental condition during holidays or punctual absences. 3) We will over-recruit participants (see Statistical power of the study). The blocked randomized design accounts for the main baseline variables that could affect the outcomes (see the section Study Design for details).

To keep attrition as low as possible, we conceived 2 attractive interventions for our patient population. We can verify and compare the motivation within the 2 groups to some extent by means of the Homework questionnaire.

For each therapist, a replacement is foreseen in the case of absence due to illness. In case of punctual absence of therapists or patients, interventions will be rescheduled as much as possible.

### Withdrawal and discontinuation

As mentioned in the informed consent form, patients can withdraw from the study at any time without providing a motivation.

If there is a suspicion of sudden cognitive impairment or the start of dementia, memory centers' professional medical doctors will validate or refute this suspicion. Whether patients may pursue interventions, if they so wish, will be determined on an individual basis by those specialists in agreement with the principal investigator of the study.

In the case of withdrawal, in the file containing the code and personal information of the patient, the date and motivation (or absence of motivation) will be mentioned, and the data of this patient will be excluded from the analyses.

### Measurements and Procedures

The research team will collect the following measures:

### Before the interventions

1. Questionnaires and information (sent to the home of the interested and fit to participate patient before the first lab visit), for details, see Supplementary Table 1**)**
  - Declaration of informed consent
  - A summary information sheet
  - Demographic questionnaire (age, sex, education level, socioeconomic status, chronic diseases, comorbidities, hearing, medication, etc.).
  - Cognitive Reserve Index Questionnaire (CRIq) [136].
  - The Amsterdam Instrumental Activity of the Daily Living Questionnaire (Short Version; (A-IADL-Q(SV)) [97] – 25 min.
  - Music education questionnaires, one elaborated in the GEMMI lab (Geneva Musical Minds lab) & the validated Gold-MSI (Goldsmiths Musical Sophistication Index) [137]
  - Godin-Shephard leisure-time physical activity questionnaire [138]
  - Major Life Events questionnaire, elaborated in the GEMMI lab
2. First laboratory visit: the team's clinical psychologist meets with the participant to explain the details of the study, and after countersigning the informed consent, verifies the participant's eligibility:
  - All questionnaires are completed
  - The Edinburgh handedness inventory (short form) [132], confirms right-handedness.
  - MRI Safety Questionnaire, provided by the BBL, determines if the participant is MRI-compatible.

After this final check-up of the inclusion/exclusion criteria, the informed consent form can be validated or destroyed.

### Before (t0, baseline), after 3 months (t1) and 6 months (t2) of interventions

#### A. Psychometric battery. Total duration: ~75 min. (interrupted by breaks)

1. D2-R test [88, 89]. Assesses attention, visual scanning accuracy and speed—8 min.
2. Trail making Test A & B [90]. Visuo-motor task, assesses selective visual attention, processing speed and mental flexibility (Shifting)—5 min.
3. Go-No-Go [91]. Inhibition task—5 min.
4. Purdue Pegboard [94]. Evaluates visuo-manual skills and bimanual coordination—10 min.
5. The Clock Drawing test [93] Evaluates apraxia and fine motor control and cognitive functions (visuo-construction, memory, spatial perception/representation, executive functions)—5 min.
6. Laterality recognition test [96]. Assesses right/left judgements of body parts presented by photos on a tablet), evaluates the representation of the body schema- 10 min.
7. Unipedal balance test [95] – < 5 min. Evaluates whole body balance.
8. ERQ: Emotion Regulation Questionnaire [121]—10 min.
9. WHOQOL-BREF [98]—5 min. Assesses well-being
10. COGTEL [79, 80]. Provides a main (weighted) score of core cognitive function -10 min.

Nota bene: We will measure the COGTEL first during the prescreening (Supplementary Table 1, PRE-SCREENING STEP 3), then at t1 (3 months) and t2 (6 months) within the psychometric battery.

The Amsterdam Instrumental Activity of the Daily Living Questionnaire (Short Version; (A-IADL-Q(SV)) [97] – 25 min will be first filled in at prescreening, then sent by post at t1 and t2 (filled in with the caregiver, then verified in the laboratory).

A detailed list of the different psychometric measurements is provided in Supplementary Table 2.

#### B. (f)MRI measurements; Total duration ~ 45 min + 15–30 min. of installation/preparation

1. Grey matter volume assessment [139] (MP2RAGE (Magnetization-Prepared 2 Rapid Acquisition Gradient Echoes)—8 min.
2. White matter assessment, allowing to compute i.a. Fractional Anisotropy (FA) for evaluating white matter integrity reflecting structural connectivity [140] (Diffusion Tensor Imaging, DTI)—7 min.
3. Resting-state functional MRI, allows to measure activity in the Default Mode Network (DMN) reflecting global functional connectivity of the brain [141]; (Echo-planar imaging, EPI)—10 min.
4. Task-related brain activity (task-fMRI). Letter n-back visual working memory task (2- (EPI)—15–20 min. (see fMRI n-back visual working memory task below).

The MRI measurements do not require any special preparation (such as having to drink or be injected with contrast materials or radioactive dye). Acquisitions are performed on a 3 T (Tesla) whole body Siemens Trio system (Siemens TIM-TRIO, Erlangen, Germany) in Geneva and on a 3 T whole body Siemens Prisma scanner (Siemens Prisma, Erlangen, Germany) in Lausanne.

We adapted the default sequences' parameters, opting for a compromise between a good signal-to-noise ratio and a minimum amount of time. The Siemens MRI protocols are optimally synchronized between the TRIO and PRISMA scanners at both sites. For the functional acquisitions, we adopted a multiband echo planar imaging fMRI with an accelerator factor of 3, allowing to reduce the repetition time to 1.350 s, which represents a good temporal resolution for fMRI (see Supplementary Table 3). Following these choices, we could reduce the total duration of the MRI scanning to 45 min, which is important in the context of our MCI patient population.

A detailed list of the different MRI measurements (*n* = 4) and main protocol parameters are provided in Supplementary Table 3.

### fMRI n-back visual working memory task

This task is an alternation between 2-back, 1-back and 0-back working memory blocks of trials using uppercase letters. We adapted it from the design developed by Migo et al. 2015 [92], a neuroimaging study conducted on amnestic MCI patients. Blocked designs are optimal for short sessions because they are associated with high activation detection power [142, 143]. This task is simple, validated and widely used in psychology, moreover, MCI patients performed it successfully [92], thus ensuring the feasibility of the task allowing comparison with the literature. Yet, we will perform a behavioral training before the fMRI session, to ensure comprehension.

**Fig. 1:**
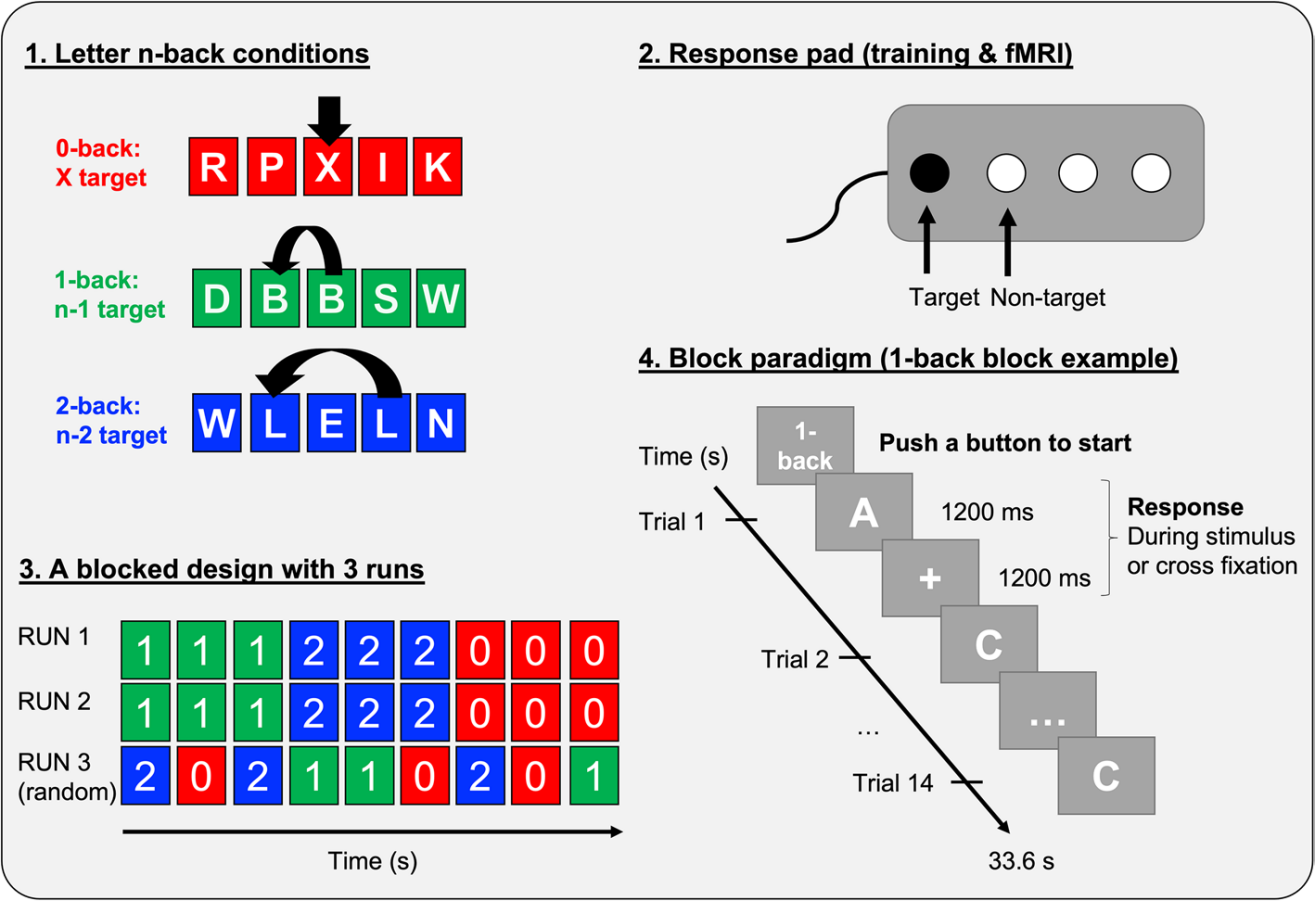
fMRI letter-n-back task: conditions and experimental design. 1. Explanation of the 3 conditions. The arrow indicates the target letter. 2. Schematic representation of the response pad used during training and MRI indicating the buttons to push for target and non-target letters. 3. Design of the 3 runs used during the task. 4. Description of the event sequence during a 1-back block. Other conditions follow the same timing

Letters will appear in series in the center of the screen. Patients are instructed to identify target letters under 3 conditions associated with an increasing load of working memory (see Fig. 1 for a complete description of the task and its paradigm). The 0-back condition is the baseline condition: patients compare the letters scrolling on the screen with one single target letter. In the 1-back condition, the target letter is the previous letter from the series (immediate repetition, e.g. “R, R “). In the 2-back condition, the target letter is the one presented 2 trials back (a repeated letter separated by another letter, e.g. “ R, A, R “).

The task will include 3 runs of 9 blocks composed of 14 letters (3 target and 11 non-target letters; 9 blocks of each condition; total duration: 15–20 min). The first 2 runs will follow a fixed order in the block presentation, whereas the last run follows a randomized block order (conditions/blocks will alternate). Instructions on the type of block (0-, 1- or 2-back condition) are presented on the screen before each block. Presentation will start when the patient is ready (he/she will push a button). Within a block, each letter is presented for 1.2 s, followed by a 1.2 s fixation cross (interstimulus interval) until the next letter appears. The patient can respond during the full 2.4 s of letter and fixation cross presentation. Stimulus presentation and interstimulus interval durations were selected upon pilot experiment results in healthy elderly and MCI patients.

## Statistical analysis plan and sample size calculation

### Basic statistical analyses

First we will use linear mixed model equations (lme) using the open-source software R [144] to evaluate the longitudinal effects for all variables separately comparing both experimental groups to the control group and to each other. These lme are flexible and powerful statistical models that consider several levels of clustering, continuous, ordinal and categorical explanatory variables. They support unbalanced data and allow to integrate demographic data into the models. Our 2 patient groups may turn out unbalanced due to attrition. Moreover, because we are interested in development, we will incorporate the baseline scores in the models. This will allow correcting for differences between the groups at baseline.

We will also compare those results with more traditional multivariate analyses of covariance.

If the data are not normally distributed, and if data transformation cannot cope with this non-normality, we will either use robust analyses (weighting outliers or other influential observations) or use non-parametric approaches.

In testing multiple contrasts, adjustments of the alpha criterion for multiple comparisons will be applied.

Concerning the primary objective, the analysis will consist in evaluating increase or less decrease (experimental group 1 or 2 vs. the control group) of the total weighted score at the COGTEL test [79, 80] at t1 and t2 compared to baseline and to the passive control group, by means of lme and MANCOVA analyses, using a two-sided alpha criterion of 0.05.

### Advanced multivariate statistical analyses

Finally, we will perform an exploratory analysis of the data using a multivariate data-driven approach. Analyzing separately behavioral data and several kinds of brain imaging data, cross-sectionally or over time, provides valuable information on these specific data. Additionally, combining diverse behavioral and different kinds of brain data within data-driven multivariate analyses may unravel hidden "covert" relationships [145, 146]. Specifically, we will perform multivariate data-driven analyses using multimodal Independent Component Analysis (ICA) [145, 146] and Partial Least of Squares (PLS) [147, 148] which have both been used successfully on multimodal brain imaging data.

Two methods will be used:

1) Joint, fusion, and linked ICA [149, 150] represent several blends of multimodal extensions of ICA that allow combining different types of data such as functional and structural neuroimaging data. The results of these analyses should reveal interactions between different types of information and consequently allow us to investigate differences between our groups / experimental conditions (interventions) [145, 149]. In addition, we will also decompose the fMRI data into functional brain networks using innovation-driven co-activation patterns (iCAPs), a recent approach of dynamic functional connectivity [151] that that can quantify temporal interactions between different networks.

2) PLS is another powerful technique that can identify components of multivariate relationships between imaging and behavioral data [148, 152], notably unified PLS correlation models [148]. This PLS technique allows revealing hidden relationships between all battery tests and MRI measurements over time and potentially show whether these relationships differ between the groups (machine-learning). We will also use PLS regression models to fit the best interventional neurobehavioral model for predicting the rehabilitation of elderly on a specific behavioral outcome i.e. evolution of the working memory performance. Implementations of these approaches are publicly available in addition to several in-house extensions that allow using them in the most flexible way, including machine-learning approaches [153, 154].

These advanced analyses will be performed in close collaboration with Prof. D. Van de Ville (computer scientist), professor of bioengineering at the EPFL (École polytechnique fédérale de Lausanne) and the University of Geneva and internationally recognized for his expertise in advanced techniques of MRI-analysis, specifically of resting-state and morphometric changes. Prof. Van De Ville and Prof. James worked together for many years.

### Statistical power of the study

We performed a statistical power analysis for sample size estimation, based on the main outcome of this project, a significant group improvement at the COGTEL test. Data used for this analysis stem from a study on MCI patients by Biasutti et Mangiacotti (2018 [62], comparing musical instrumental practice (*n* = 15) to soft gymnastic activities (*n* = 14). In this study, the main outcome was a change over time in the MMSE score. Between baseline and the end of their twice weekly 3-month interventions, similar to the interventions in the current study, the MMSE performance did not change in the soft gymnastics group whereas it improved significantly in the musical instrumental practice group (F(1,33) = 13.906; *p* < 0.001; pη2 = 0.296). Such a partial η2 value corresponds to Cohen's effect size f of 0.65 which is considered large [155]. With an alpha = 0.05 and power = 0.80, projected sample size required for this effect size is *n* = 12 (GPower 3.1) [156].

The MMSE being highly correlated to the COGTEL (r = 0.93) [80], our proposed sample size of *n* = 16 per group (or *n* = 12 considering an attrition rate of 20%) seems reasonable for observing a significant COGTEL effect.

We recruit in two university hospital memory clinics; sample size was in the COVID context also determined according to their databases and recommendations, to ensure feasibility, but we will respect our power calculation above.

In general, it is difficult to recruit older adults for interventional neuroimaging studies. MRI measurements represent an impediment for older adults, even if they are mentally fit. Those studies therefore suffer from under recruitment. Even with fewer healthy older individuals than the number of patients suggested in the current protocol, many authors could still publish significant findings [157–163].

### Attrition / Drop-outs

A total of 16 volunteers will be recruited for each of the experimental patient groups and the control group. Thus, the intended total number of participants will be 48. Given the unclear future of the covid pandemic and the natural attrition in our patient population, our minimum aim is a total of at least 12 participants in each group at the completion of the six-month interventions and measurements, which corresponds to our power analyses (see section Statistical power of the study). We maintain the 16-person target for the control group. Thus, we predict a 20% attrition rate among the patients and will over-recruit 32 participants. We will not be able to compensate for dropouts because the interventions are ongoing.

## Data monitoring and quality assurance

Data acquisition and quality is continuously monitored by the experimenters for each patient. A second quality check is performed on the server of the team where all data will be backed up (see Declarations**,** Availability of data and materials) and analyzed by the scientific collaborator (postdoc level) of the research team.

A third checkup is performed by an independent clinical Data Manager (scientific collaborator at PhD level) at the HEdS, who will regularly check all steps of the protocol as accepted by the ethical commissions (randomization, anonymization, inclusion/exclusion criteria, signed informed consent forms, questionnaires, data collection). All source data, documents and outcome data will be available to the monitor, without restriction.

The research team, including a Master student, will meet twice every month to discuss the progress of the study and encountered problems. Any urgent problem will be discussed immediately to assure the quality of the data collection (for instance sudden cognitive deterioration of a patient).

We will report reasons for the withdrawal of individuals for each randomization group and for the passive control group. Exact information on missing data at each time point will be reported in all publications. Missing data will be processed in the analyses with cutting edge means (for instance using the regularized iterative Principal Component Analysis (PCA) algorithm developed by Josse, Husson and Pagès (2009) [164], implemented in the R package missMDA).

## Discussion

This RCT combines comprehensive psychometric data with different structural and functional brain imaging measurements in MCI patients. Compared to a passive control group, the combined data serve comparing long-term effects of music practice and psychomotor practice interventions on beginning pathological cognitive decline.

In the context of our study, we specifically designed 2 innovative interventions (music practice and psychomotor practice) tailored for our population of MCI patients. Very recently, Biasutti and Mangiacotti [62, 63] showed encouraging results in MCI patients at the psychometric level, comparing a musical practice group to an active control group that received a soft gymnastics intervention, with an improvement of MMSE scores in the musical practice group only.

Replicating this finding for our main outcome, the COGTEL weighted score, which is highly correlated to the MMSE but more fine-grained (no ceiling effect for healthy participants), would represent a major gain for the science community and the development of non-pharmaceutical interventions aiming to enhance cognitive functioning in MCI patients and other elderly patient populations with cognitive decline. Moreover, in contrast to the soft gymnastics offered as an active control group in the study by Biasutti and Mangiacotti [62], we apply a second experimental condition, with already validated therapeutical benefits, and additionally a passive healthy control group allowing to control for age-related natural brain atrophy and test–retest effects of the psychometric battery and fMRI tasks.

The second experimental condition, psychomotor practice, may offer specific and different benefits as compared to the music practice group (see the section Specific outcomes according to each intervention). Contrasts and conjunction analyses will allow to examine overlapping effects of both interventions as well as their potential specific benefits.

Compared to Biasutti and Mangiacotti [62] the current study will acquire more data points, over a longer period of time, and given the multimodality of our data collection that integrates behavioral and diverse functional and structural brain data, we will be able to further explore the potential plasticity of cognitive and sensorimotor behavior, brain structure and function and their relationships following the experimental interventions in MCI patients.

We will respect this protocol closely, provided that the COVID-19 pandemic allows for patient recruitment and adherence during this period. Dates are subject to change as the sanitary situation evolves.

### Broader impact

At present, the only conclusion from clinical and translational research is that dementia, of which Alzheimer's disease is one, is not curable. Despite significant progress made in the early detection and, consequently, in the delay and reduction of symptoms, the progression of the disease, once started, is irreversible. Therefore, approaches that can delay, diminish or even temporarily overcome brain decline, especially at early stages, are crucial.

In an ideal situation, these therapies would be simple to incorporate into activities of daily living activities, joyful and stimulating, and suitable to be sustained over extended periods of time (several years). In an elderly population, a lack of motivation frequently thwarts the success of exercise regimens.

If the experimental interventions in the current study show positive influences on cognitive and sensorimotor performances of MCI patients, this study may trigger the development and implementation of targeted non-medical long-term interventions for different types of elderly patients in the context of population aging and lifespan development and extend autonomy, serve public health, and therefore reduce health costs.

### Innovative aspects

- Develop unconventional low-cost ecological behavioral group interventions aiming to improve cognitive and sensorimotor function and induce brain plasticity in MCI patients.
- Potentially demonstrate that the slope of decline in the fragile population of MCI patients can be postponed or reversed via music and or psychomotor practice, increasing the autonomy and well-being of these patients.
- Comparisons of 2 distinct experimental "real-life" interventions over 6 months to a control group may shed light on the specific effects of those interventions in the context of pathological age-related decline.
- By incorporating brain data, we can gain a more comprehensive understanding of these early-stage age-related mental disorders and their potential development.
- Merging comprehensive psychometric and structural and functional brain data within advanced multivariate analyses may unravel hidden relationships and shed new light on cognitive decline (see the section Advanced multivariate statistical analyses).

## Supplementary information

**Additional file 1: Supplementary Table 1. **Prescreening procedure1 to verify inclusion/exclusion criteria.

**Additional file 2: Supplementary Table 2.** Behavioral Battery (psychometric tests)Measured at all 3 time points: t0: baseline; t1: 3 months; t2: 6 months COGTEL & A-IADL-Q(SV) applied before t0, then at t1 and t2.

**Additional file 3: Supplementary Table 3.** MRI1 measurements Measured at all 3 time points: t0: baseline; t1: 3 months; t2: 6 months.

## Data Availability

Collected data will be anonymized and stored at a secured server hosted in a secured datacenter at the HES-SO Geneva, accessible to a limited number of members of the research team, protected by a secure password. The server will also be used for all analyses. As soon as the data collection will be completed, all data and all other documents will also be stored at YARETA, a FAIR digital solution for long-term preservation of research data for all Geneva Universities (https://yareta.unige.ch). The datasets generated during the current longitudinal study will not be publicly available immediately, only after the team published the main outcomes.

## Abbreviations

(f)MRI: (functional) Magnetic Resonance Imaging
A-IADL-Q(SV): The Amsterdam Instrumental Activity of the Daily Living Questionnaire (Short Version)
BBL: Brain and Behaviour Laboratory
CCER: Commission cantonale d’éthique de la recherche sur l'être humain de Genève
CER-VD: Commission cantonale d'éthique de la recherche sur l'être humain de Vaud
CHUV: Centre Hospitalier Universitaire Vaudois / Lausanne University Hospital
COGTEL: Cognitive Telephone Screening Instrument
COVID-19: Pandemic of the virus named SARS-CoV-2 (2019-nCoV)
CRIq: Cognitive Reserve Index questionnaire
DMN: Default Mode Network
DTI: Diffusion Tensor Imaging
EPI: Echo-Planar Imaging
ERQ: Emotion Regulation Questionnaire
FA: Fractional Anisotropy
FOV: Field Of View
GE: Geneva (Switzerland)
GEMMI lab: Geneva Musical Minds laboratory
Gold-MSI: The Goldsmiths Musical Sophistication Index
HADS: Hospital Anxiety and Depression Scale
HES-SO: Haute École Spécialisée de Suisse occidentale / University of Applied Sciences and Arts Western Switzerland
HEdS-GE: Haute école de santé de Genève HES-SO / Geneva School of Health Sciences
HUG: Hôpitaux Universitaires de Genève / Geneva University Hospitals
ICA: Independent Component Analysis
iCAPs: innovation-driven Co-Activation Patterns
lme: Linear mixed models equations
LMC-CHUV: Centre Leenaards de la Mémoire au Centre Hospitalier Universitaire Vaudois / Leenaards Memory Center at the Lausanne University Hospital
LREN: Laboratoire de recherche en neuroimagerie / Laboratory for Research in Neuroimaging
MANCOVA: Multivariate analysis of covariance
MC-HUG: Centre de la Mémoire des Hopitaux Universitaires de Genève / Memory Center of the Geneva University Hospitals
MCI: Mild Cognitive Impairment
MoCA: Montreal Cognitive Assessment
MP2RAGE: Magnetization-Prepared 2 Rapid Acquisition Gradient Echoes
MMSE: Mini-Mental State Examination
MRI: Magnetic Resonance Imaging
PCA: Principal Component Analysis
PLS: Partial Least of Squares
RCT: Randomized Control Trial
T: Tesla
t0: Baseline measurement
t1: 3-Month mid-term measurement
t2: 6-Month post-intervention measurement
WHOQOL-BREF: World Health Organization Quality of Life-BREF questionnaire (BREF for abbreviated)

## Footnotes

1 Cognitive Telephone Screening Instrument. This test can be applied by telephone or face-to-face. We will use the latter approach.

2 The Clock drawing test pertains to both cognitive and sensorimotor functions, as it associates the evaluation of fine motor control with cognitive functions (visuo-construction, memory, spatial perception, executive functions).

3 Hôpitaux Universitaires de Genève / Geneva University Hospitals.

4 Centre Hospitalier Universitaire Vaudois / Lausanne University Hospital.

5 Patients participating in the interventions declare aiming to perform homework at least 30 min per day, 4 times per week, not to miss more than maximum 4 courses (on 40 in total) and announce absences and holidays well in advance with reasons, and not to engage in any supplementary activities similar to those proposed in this study.
