# Supplementary material for: Musical and psychomotor interventions for cognitive, sensorimotor, and cerebral decline in patients with Mild Cognitive Impairment (COPE): a study protocol for a multicentric randomized controlled study
Source: BMC Geriatr. 2023 Feb 6;23:76. doi: 10.1186/s12877-022-03678-0 (PMC9900212; doi:10.1186/s12877-022-03678-0)
Supplement: Supplementary file 1 — Additional file 1: Supplementary Table 1. Prescreening procedure1 to verify inclusion/exclusion criteria. [file 12877_2022_3678_MOESM1_ESM.pdf]

Supplementary Table 1: Prescreening procedure[1] to verify inclusion/exclusion criteria

**PRESCREENING STEP 1**	Action	Data collection
Hospital-provided data check	Acquired & transmitted by the HUG & the CHUV	Clinical functional and structural MRI<br>Behavioral data: Mini Mental State Examination (MMSE) **[1]**, MoCA **[2],** Hospital Anxiety and Depression Scale (HADS) **[3]**
First contact with research team	Telephone call; decision to proceed to Pre-screening Step 2	Age – gender – musical and recent physical activity history – general health – native language – MRI compatibility – overall availability – contact information
**PRESCREENING STEP 2**		
Summary information sheet & declaration of informed consent	Send/receive by post	Read the information sheet, read the declaration of informed consent and sign it before filling in any of the following questionnaires.
Demographic questionnaire[2]	Send/receive by post	Laboratory-elaborated questionnaire (GEMMI lab): detailed information on **languages**; **handedness; physical capability** to **come to the interventions; health**: hospitalizations (causes), locomotion, vision (correction), audition (hearing aids), height & weight[2], sleep quality; cardiovascular & neurological diseases, hypertension, diabetes, arthrosis, arthritis, rheumatism, bone fractures, thyroid problems, memory problems, other health problems; **fortuitous findings**: who (treating physician or patient) to contact; **smoking** habits over the lifespan; **alcohol consumption** over the lifespan; present **medication**; other **drug use**; **sociodemographic information:** age, gender, nationality, civil status, siblings, children, highest level of education (5 categories), main professional engagements, gross monthly household income[3] (5 categories); **MRI compatibility:** claustrophobia, implants, etc.; **Interest for the study:** motivation
Cognitive Reserve Index Questionnaire[4] **[4]**	Send/receive by post	The CRIq includes demographic data and items grouped into three sections: education, working activity and leisure time, each of which returns a subscore
A-IADL-Q(SV) **[5]**	Send/receive by post	The Amsterdam Instrumental Activity of the Daily Living Questionnaire (Short Version; (A-IADL-Q(SV)), examines proficiency in daily living activities
Music education questionnaires	Send/receive by post	Musical history laboratory-elaborated questionnaire (GEMMI lab): amount and intensity of music listening and extracurricular music training: solfege/theory, instrumental training, choir singing, intracurricular music training in school<br>Goldsmiths Musical Sophistication Index (Gold-MSI) **[6]**: self-report inventory for individual differences in musical sophistication (ability to engage with music)
Godin-Shephard leisure-time physical activity questionnaire **[7]**	Send/receive by post	Adapted to cover physical activities in different parts of life (12-25 y.o., last 5 years and last year)
Laboratory-elaborated questionnaire on major life events	Send/receive by post	Self-report inventory on stressful events that may influence cognitive functioning, covering 6 time periods (12-25 y.o., 25-55 y.o, 55-64 y.o.; last 10 years, last 5 years and last year); GEMMI lab
**PRESCREENING STEP 3**		
First laboratory visit<br><br><br>- Psychometric tests<br>i. Right-handedness **[8, 9]**<br>ii. COGTEL[8] **[10, 11]**<br>iii. International Matrix Test (Oldenburg)[6] [12]	Research team member countersigns declaration of informed consent<br>Verify/complete questionnaires of Step 2<br><br>Pass 3 psychometric tests	i. Edinburgh Handedness Inventory (revised) to evaluate degree of right handedness; ii. Measure gross overall cognitive level and check for potential (mild) cognitive decline (COGTEL); iii. Check for major hearing impairment.

Footnotes Table 2

1. Outliers (based on cut-off scores and or other exclusion criteria) for the tests of Pre-screening Step 1, 2 & 3 are excluded progressively from the study; the declaration of informed consent will be destroyed
2. Allows to compute the Body Mass Index (BMI), obese people are excluded (BMI > 30), as obesity impacts on brain (shrinkage) **[13]**
3. Adapted to Switzerland and France
4. Cognitive reserve is the resilience to neuro-pathological damage in older adults **[14].**
5. The COGTEL test provides a global measure of cognitive function, it comprises 6 subtests covering prospective memory, short- and long-term verbal memory, working memory (digit span), verbal fluency and inductive reasoning.
6. The first stage of the International Matrix Test (Oldenburg) assesses intelligibility and understanding of speech and allows to check for major hearing impairment

1. Folstein MF, Folstein SE, McHugh PR: **"Mini-Mental State": A practical method for grading the cognitive state of patients for the clinician**. *J Psychiatr Res* 1975, **12**:189–198.
2. Trzepacz PT, Hochstetler H, Wang S, Walker B, Saykin AJ, Alzheimer's Disease Neuroimaging I: **Relationship between the Montreal Cognitive Assessment and Mini-mental State Examination for assessment of mild cognitive impairment in older adults**. *BMC Geriatr* 2015, **15**:107.
3. Zigmond AS, Snaith RP: **The hospital anxiety and depression scale**. *Acta Psychiatr Scand* 1983, **67**(6):361-370.
4. Nucci M, Mapelli D, Mondini S: **Cognitive Reserve Index questionnaire (CRIq): a new instrument for measuring cognitive reserve**. *Aging clinical and experimental research* 2012, **24**(3):218-226.
5. Jutten RJ, Peeters CFW, Leijdesdorff SMJ, Visser PJ, Maier AB, Terwee CB, Scheltens P, Sikkes SAM: **Detecting functional decline from normal aging to dementia: Development and validation of a short version of the Amsterdam IADL Questionnaire**. *Alzheimers Dement (Amst)* 2017, **8**:26-35.
6. Mullensiefen D, Gingras B, Musil J, Stewart L: **The musicality of non-musicians: an index for assessing musical sophistication in the general population**. *PloS one* 2014, **9**(2):e89642.
7. Amireault S, Godin G: **The Godin-Shephard leisure-time physical activity questionnaire: validity evidence supporting its use for classifying healthy adults into active and insufficiently active categories**. *Percept Mot Skills* 2015, **120**(2):604-622.
8. Oldfield RC: **The assessment and analysis of handedness: the Edinburgh inventory**. *Neuropsychologia* 1971, **9**(1):97-113.
9. Veale JF: **Edinburgh handedness inventory–short form: a revised version based on confirmatory factor analysis**. *Laterality: Asymmetries of Body, Brain and Cognition* 2014, **19**(2):164-177.
10. Ihle A, Gouveia ER, Gouveia BR, Kliegel M: **The Cognitive Telephone Screening Instrument (COGTEL): A Brief, Reliable, and Valid Tool for Capturing Interindividual Differences in Cognitive Functioning in Epidemiological and Aging Studies**. *Dement Geriatr Cogn Dis Extra* 2017, **7**(3):339-345.
11. Breitling LP, Wolf M, Muller H, Raum E, Kliegel M, Brenner H: **Large-scale application of a telephone-based test of cognitive functioning in older adults**. *Dementia and geriatric cognitive disorders* 2010, **30**(4):309-316.
12. Kollmeier B, Warzybok A, Hochmuth S, Zokoll MA, Uslar V, Brand T, Wagener KC: **The multilingual matrix test: Principles, applications, and comparison across languages: A review**. *International Journal of Audiology* 2015, **54**(sup2):3-16.
13. Hamer M, Batty GD: **Association of body mass index and waist-to-hip ratio with brain structure: UK Biobank study**. *Neurology* 2019, **92**(6):e594-e600.
14. Tucker AM, Stern Y: **Cognitive reserve in aging**. *Current Alzheimer research* 2011, **8**(4):354-360.
