# Supplementary material for: Musical and psychomotor interventions for cognitive, sensorimotor, and cerebral decline in patients with Mild Cognitive Impairment (COPE): a study protocol for a multicentric randomized controlled study
Source: BMC Geriatr. 2023 Feb 6;23:76. doi: 10.1186/s12877-022-03678-0 (PMC9900212; doi:10.1186/s12877-022-03678-0)
Supplement: Supplementary file 2 — Additional file 2: Supplementary Table 2. Behavioral Battery (psychometric tests)Measured at all 3 time points: t0: baseline; t1: 3 months; t2: 6 months COGTEL & A-IADL-Q(SV) applied before t0, then at t1 and t2. [file 12877_2022_3678_MOESM2_ESM.pdf]

Supplementary Table 2: Behavioral Battery (psychometric tests)
Measured at all 3 time points: t0: baseline; t1: 3 months; t2: 6 months
COGTEL & A-IADL-Q(SV) applied before t0, then at t1 and t2

**BASIC COGNITION**		
Visual processing speed & Attention	d2-R test	Brickenkamp and Zillmer 1998 [1, 2]
Visual processing speed, Shifting & Hand motor skill	Trail Making Test A & B	Strauss et al., 2006 [3]
**EXECUTIVE CONTROL**		
Inhibition	Go/No-Go	Enge et al., 2014 [4]
[Visual working memory (2-back)	fMRI in-scanner task	Migo et al, 2015 [5]]
**AUDITIVE COGNITION**		
Speech-in-noise perception	International Matrix Test (Oldenburg)	Kollmeier et al., 2015 [6]
**PERCEPTUAL MOTOR SKILL**		
Visuo-manual aptitude	Purdue Pegboard	Tiffin and Asher, 1948 [7]
Visuo-construction, fine motor control, memory and executive functions	The Clock Drawing Test	Aprahamian et al., 2009 [8]
Assessing right/left judgements of photos of body parts on a tablet	Laterality recognition test (evaluates the representation of the body schema)	Williams at al., 2019 [9]
Whole body balance	Unipedal balance test	Bohannon and Tudini, 2018 [10]
**OTHER**		
Well-being	WHOQOL-BREF*	WHO, 1996 [11]
Autonomy in daily living activities	A-IADL-Q(SV)**	Jutten et al., 2017 [12]
Emotional Regulation	ERQ	Christophe et al, 2009 [13]
**MAIN SCORE OF COGNITIVE FUNCTION**	COGTEL test	Breitling et al., 2010; Ihle et al., 2017; [14, 15]

*World Health Organization Quality of Life-BREF (BREF for abbreviated)
** The Amsterdam Instrumental Activity of the Daily Living Questionnaire (Short Version)

1. Brickenkamp R, Zillmer E: **The d2 Test of Attention. 1998**. *Goettingen, Hogrefe* 1998.
2. Brickenkamp R, Schmidt-Atzert L, Liepmann D: **Test d2-Revision: Aufmerksamkeits- und Konzentrationstest**: Hogrefe Göttingen; 2010.
3. Strauss PPE, Strauss E, Sherman NAAPDPCNEMS, Sherman EMS, Spreen O: **A Compendium of Neuropsychological Tests: Administration, Norms, and Commentary**: Oxford University Press; 2006.
4. Enge S, Behnke A, Fleischhauer M, Kuttler L, Kliegel M, Strobel A: **No evidence for true training and transfer effects after inhibitory control training in young healthy adults**. *J Exp Psychol Learn Mem Cogn* 2014, **40**(4):987-1001.
5. Migo EM, Mitterschiffthaler M, O'Daly O, Dawson GR, Dourish CT, Craig KJ, Simmons A, Wilcock GK, McCulloch E, Jackson SH *et al*: **Alterations in working memory networks in amnestic mild cognitive impairment**. *Neuropsychol Dev Cogn B Aging Neuropsychol Cogn* 2015, **22**(1):106-127.
6. Kollmeier B, Warzybok A, Hochmuth S, Zokoll MA, Uslar V, Brand T, Wagener KC: **The multilingual matrix test: Principles, applications, and comparison across languages: A review**. *International Journal of Audiology* 2015, **54**(sup2):3-16.
7. Tiffin J, Asher EJ: **The Purdue Pegboard: Norms and studies of reliability and validity**. *Journal of Applied Psychology* 1948, **32**:234–247.
8. Aprahamian I, Martinelli JE, Neri AL, Yassuda MS: **The Clock Drawing Test: A review of its accuracy in screening for dementia**. *Dement Neuropsychol* 2009, **3**(2):74-81.

9. Williams LJ, Braithwaite FA, Leake HB, McDonnell MN, Peto DK, Moseley GL, Hillier SL: **Reliability and validity of a mobile tablet for assessing left/right judgements**. *Musculoskeletal Science and Practice* 2019, **40**:45-52.
10. Bohannon RW, Tudini F: **Unipedal balance test for older adults: a systematic review and meta-analysis of studies providing normative data**. *Physiotherapy* 2018, **104**(4):376-382.
11. Organization WH: **WHOQOL-BREF: introduction, administration, scoring and generic version of the assessment: field trial version, December 1996**. In.: World Health Organization; 1996.
12. Jutten RJ, Peeters CFW, Leijdesdorff SMJ, Visser PJ, Maier AB, Terwee CB, Scheltens P, Sikkes SAM: **Detecting functional decline from normal aging to dementia: Development and validation of a short version of the Amsterdam IADL Questionnaire**. *Alzheimers Dement (Amst)* 2017, **8**:26-35.
13. Christophe V, Antoine P, Leroy T, Delelis G: **Évaluation de deux stratégies de régulation émotionnelle: la suppression expressive et la réévaluation cognitive**. *European review of applied psychology* 2009, **59**(1):59-67.
14. Breitling LP, Wolf M, Muller H, Raum E, Kliegel M, Brenner H: **Large-scale application of a telephone-based test of cognitive functioning in older adults**. *Dementia and geriatric cognitive disorders* 2010, **30**(4):309-316.
15. Ihle A, Gouveia ER, Gouveia BR, Kliegel M: **The Cognitive Telephone Screening Instrument (COGTEL): A Brief, Reliable, and Valid Tool for Capturing Interindividual Differences in Cognitive Functioning in Epidemiological and Aging Studies**. *Dement Geriatr Cogn Dis Extra* 2017, **7**(3):339-345.
