# Supplementary material for: Musical and psychomotor interventions for cognitive, sensorimotor, and cerebral decline in patients with Mild Cognitive Impairment (COPE): a study protocol for a multicentric randomized controlled study
Source: BMC Geriatr. 2023 Feb 6;23:76. doi: 10.1186/s12877-022-03678-0 (PMC9900212; doi:10.1186/s12877-022-03678-0)
Supplement: Supplementary file 3 — Additional file 3: Supplementary Table 3. MRI1 measurements Measured at all 3 time points: t0: baseline; t1: 3 months; t2: 6 months. [file 12877_2022_3678_MOESM3_ESM.pdf]

Supplementary Table 3: MRI[1] measurements
Measured at all 3 time points: t0: baseline; t1: 3 months; t2: 6 months

TYPE OF MRI MEASUREMENT / SCAN	Main technical details of the Siemens protocols
**Grey matter volume assessment [1]**	MP2RAGE[2]; duration: 8.22 minutes; voxel size: 1 mm isotropic; 176 slices; FOV[3]: 256x240x176 mm; TR[4]/TE[5]: 5000/2.98 ms; TI1[6]/TI2[7]: 700/2500 ms; flip angle 1/2: 4/5 degrees
**Functional MRI**<br>Letter N-back visual working memory task **[2]**	EPI[8]; duration: 20.00 minutes; voxel size: 2.5 mm isotropic; 54 slices; FOV: 210x210x135 mm; multiband accelerator factor: 3; TR/TE: 1350/31.6 ms
**Resting-state functional MRI**<br>Allows to measure activity in the Default Mode Network (DMN) reflecting global functional connectivity of the brain **[3]**	EPI; duration: 10.31 minutes; voxel size: 2.5 mm isotropic; 54 slices; FOV: 210x210x135 mm; multiband accelerator factor: 3; TR/TE: 1350/31.6 ms
**White matter assessment**<br>Allows to compute i.a. Fractional Anisotropy for evaluating white matter integrity reflecting structural connectivity **[4]**.	DTI[9]; duration 7.38 minutes; voxel size: 1.5 mm isotropic; 84 slices; FOV: 222x222x126 mm; multiband accelerator factor: 3; TR/TE: 5163/109.2 ms; b-values: 0/1500 s/mm^2; 65 directions, anterior to posterior phase encoding direction followed by a reverse direction for distortion correction; b-values: 0/ s/mm^2)

Footnotes Table 5
1. MRI: Magnetic Resonance Imaging
2. MP2RAGE: Magnetization-Prepared 2 Rapid Acquisition Gradient Echoes
3. FOV: Field Of View
4. TR: Time to Repetition
5. TE: Time to Echo
6. TI1: Inversion Time 1
7. TI2; Inversion Time 2
8. EPI: Echo Planar Imaging
9. DTI: Diffusion Tensor Imaging

1. Marques JP, Gruetter R: **New developments and applications of the MP2RAGE sequence--focusing the contrast and high spatial resolution R1 mapping**. *PloS one* 2013, **8**(7):e69294.
2. Migo EM, Mitterschiffthaler M, O'Daly O, Dawson GR, Dourish CT, Craig KJ, Simmons A, Wilcock GK, McCulloch E, Jackson SH *et al*: **Alterations in working memory networks in amnestic mild cognitive impairment**. *Neuropsychol Dev Cogn B Aging Neuropsychol Cogn* 2015, **22**(1):106-127.
3. Leonardi N, Richiardi J, Gschwind M, Simioni S, Annoni JM, Schluep M, Vuilleumier P, Van De Ville D: **Principal components of functional connectivity: a new approach to study dynamic brain connectivity during rest**. *NeuroImage* 2013, **83**:937-950.
4. Bosch B, Arenaza-Urquijo EM, Rami L, Sala-Llonch R, Junque C, Sole-Padulles C, Pena-Gomez C, Bargallo N, Molinuevo JL, Bartres-Faz D: **Multiple DTI index analysis in normal aging, amnestic MCI and AD. Relationship with neuropsychological performance**. *Neurobiol Aging* 2012, **33**(1):61-74.
